# Plant-specific calreticulin is localized in the nuclei of highly specialized cells in the pistil—new observations for an old hypothesis

**DOI:** 10.1007/s00709-024-01961-y

**Published:** 2024-06-07

**Authors:** Piotr Wasąg, Anna Suwińska, Anna Richert, Marta Lenartowska, Robert Lenartowski

**Affiliations:** 1 Department of Cellular and Molecular Biology, Faculty of Biological and Veterinary Sciences, Nicolaus Copernicus University in Toruń, Toruń, Poland; 2 Centre for Modern Interdisciplinary Technologies, Nicolaus Copernicus University in Toruń, Toruń, Poland

**Keywords:** CRT, Exchangeable Ca^2+^, *Haemanthus*, Nucleus, *Petunia*, Pistil transmitting tract

## Abstract

One of the first cellular locations of the calreticulin (CRT) chaperone in eukaryotic cells, apart from its obvious localization in the endoplasmic reticulum (ER), was the cell nucleus (Opas et al. 1991). The presence of CRT has been detected inside the nucleus and in the nuclear envelope of animal and plant cells, and a putative nuclear localization signal (NLS) in the CRT amino acid sequence has been mapped in several animal and plant species. Over the last 30 years, other localization sites of this protein outside the ER and cell nucleus have also been discovered, suggesting that CRT is a multifunctional Ca^2+^-binding protein widely found in various cell types. In our previous studies focusing on plant developmental biology, we have demonstrated the presence of CRT inside and outside the ER in highly specialized plant cells, as well as the possibility of CRT localization in the cell nucleus. In this paper, we present a detailed analysis of immunocytochemical localization of CRT inside nuclei of the pistil transmission tract somatic cells before and after pollination. We show a similar pattern of the nuclear CRT localization in relation to exchangeable Ca^2+^ for two selected species of angiosperms, dicotyledonous *Petunia* and monocot *Haemanthus*, that differ in anatomical structure of the pistil and discuss the potential role of CRT in the cell nucleus.

**Supplementary Information:**

The online version contains supplementary material available at 10.1007/s00709-024-01961-y.

## Introduction

When an ER-associated protein was first identified in eukaryotic cells (Ostwald and MacLennan 1974), no one suspected how multifunctional CRT could be. Currently, CRT is defined as ubiquitously expressed Ca^2+^-binding/buffering molecular chaperone that is evolutionarily conserved in all eukaryotes except yeasts (Michalak 2023). Molecular analysis revealed two *CRT* genes (*CRT1* and *CRT2*) in animals and at least one additional gene, *CRT3*, in plant genomes (Persson et al. 2003; Jia et al. 2008; Wasąg et al. 2022). Moreover, plant genomes contain multiple copies of *CRT* family member genes resulting from duplication events (Del Bem 2011; Wasąg et al. 2019). Initially, CRT as a soluble ER luminal protein was closely related to Ca^2+^ homeostasis/signaling and molecular chaperoning of newly synthetized proteins in the ER. This protein cooperates with another ER chaperone, the transmembrane protein calnexin (CNX), in the so-called CNX/CRT cycle responsible for the mechanism of recognition of misfolded glycoproteins. Currently, due to repeated confirmation of the occurrence of CRT in various cellular locations outside the ER, it has been implicated in multiple intra/extracellular processes correlated with apoptosis, environmental stress response, immunity, cell-to-cell communication, or sexual plant reproduction (Persson et al. 2003; Jia et al. 2009; Suwińska et al. 2017, 2022; Michalak 2023). CRT is assumed to be involved in these many processes through its two main functions: Ca^2+^ storage/mobilization and protein folding. In plant cells, in addition to the typical ER localization, CRT was detected in the cytosol (Jia et al. 2008; Wasąg et al. 2018), dictyosomes (Borisjuk et al. 1998; Navazio et al. 2002; Lenartowska et al. 2009; Hsieh and Huang 2005; Nardi et al. 2006; Lenartowski et al. 2015; Niedojadło et al. 2015; Wasąg et al. 2018), plasma membrane (Borisjuk et al. 1998; Navazio et al. 2002; Šamaj et al. 2008; Wasąg et al. 2018), cell wall/extracellular matrix (Lenartowska et al. 2009; Lenartowski et al. 2015; Luczak et al. 2015; Niedojadło et al. 2015; Wasąg et al. 2018), and plasmodesmata (Baluska et al. 1999; Bayer et al. 2004; Chen et al. 2005; Lenartowska et al. 2009; Bilska and Sowinski 2010; Christensen et al. 2010; Wasąg et al. 2018).

**Fig. 1:**
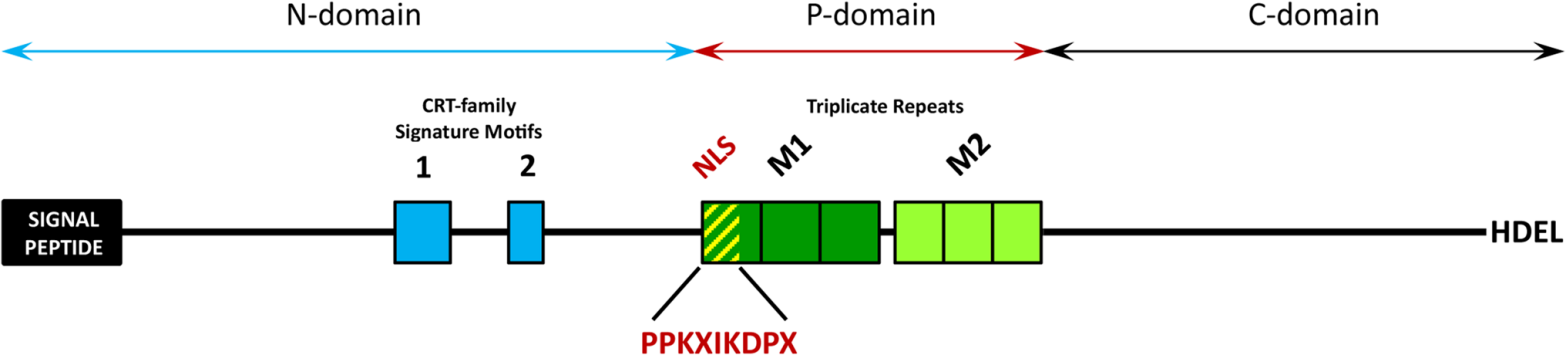
Schematic model of CRT protein. The colored two-headed arrows represent protein domains containing elements characteristic for CRT family proteins. The sequence begins with a signal peptide (black rectangular) and concludes with the ER-retention motif (HDEL). Blue boxes indicate CRT family signature motifs 1 and 2, while dark and light green boxes represent triplicate repeats M1 and M2, respectively. The NLS peptide signal (PPKXIKDPX) is marked by yellow-green rectangular, located at the start of the P-domain and partially overlapping with the M1 repeat

One of the earliest locations of CRT, both in animal and plant cells, were the cell nucleus and nuclear envelope (Opas et al. 1991). The key premise authenticating the occurrence of the CRT in this cellular compartment was the mapping of the putative NLS within the peptide sequence (Fig. 1) for different animal and plant-type cells (Michalak et al. 1992; Denecke et al. 1995; Borisjuk et al. 1998; Mushtaq et al. 2020). Nuclear localization was confirmed, among others, for rat myoblasts and epithelial cells (Opas et al. 1991), osteosarcoma cells (Dedhar et al. 1994), megakaryocytes (Iborra and Papadopoulos 2017), different developmental forms of *Trypanosoma cruzi* (Souto-Padron et al. 2004), tobacco cotyledon cells (Denecke et al. 1995), maize root cells (Napier et al. 1995), onion epidermal cells (Jia et al. 2008), and *Chara vulgaris* spermatids (Popłońska 2013), suggesting a potential involvement of CRT in gene expression, nuclear transport, stability of mRNAs, and chromatin rearrangement of nuclear proteins during spermatogenesis. Moreover, in our previous studies focusing on plant reproductive biology, we have indicated the possibility of specific localization of CRT in the nuclei of highly specialized somatic cells involved in pollen formation in the anther (Suwińska et al. 2022) or pollen tube growth in the pistil (Lenartowski et al. 2015). Since all previously published research results confirming the occurrence of CRT in cell nuclei are incidental, the nuclear localization of this protein and its function in the nucleus is still under debate.

Pollen-pistil interactions are a crucial aspect of flowering plant reproduction, involving three successive stages. Firstly, pollen grains germinate on the stigma (pollination). Subsequently, two immotile sperm cells are delivered to the embryo sac by a pollen tube (progamic phase), followed by double fertilization process in the ovary. Throughout these stages, cellular cross-talk occurs between the germinating pollen and growing pollen tubes with the cells of the pistil transmitting tract. This tract extends from the stigma through the pistil style to the ovary, constituting the natural environment for pollen tube growth in vivo (Dresselhaus and Franklin-Tong 2013). Mature pistils of angiosperms exhibit differentiation based on the anatomical structure of stigmas and pistil styles in various plant species. Wet stigmas typically have small papillae and are covered with exudate, while the dry stigmas, featuring multicellular papillate, have little or no surface secretion at the receptive stage. The anatomical organization of the style is categorized as either hollow or solid. Hollow styles feature a distinct secretion-filled canal lined by secreting epidermal cells that transverses the style. In solid styles, characteristic for many dicotyledons, a compact transmitting tissue forms a central core, including narrow secretion-filled intercellular spaces. Cells of the pistil transmitting tract exhibit secretory characteristics, with the ER, dictyosomes, and plastids as dominating organelles as well as with active cell nuclei containing large, often multiple nucleoli. During the progamic phase, these extremely metabolically active cells promote the directional growth of pollen tubes towards the ovules for fertilization, followed by programed cell death in the transmitting tract cells (Crawford et al. 2007).

**Fig. 2:**
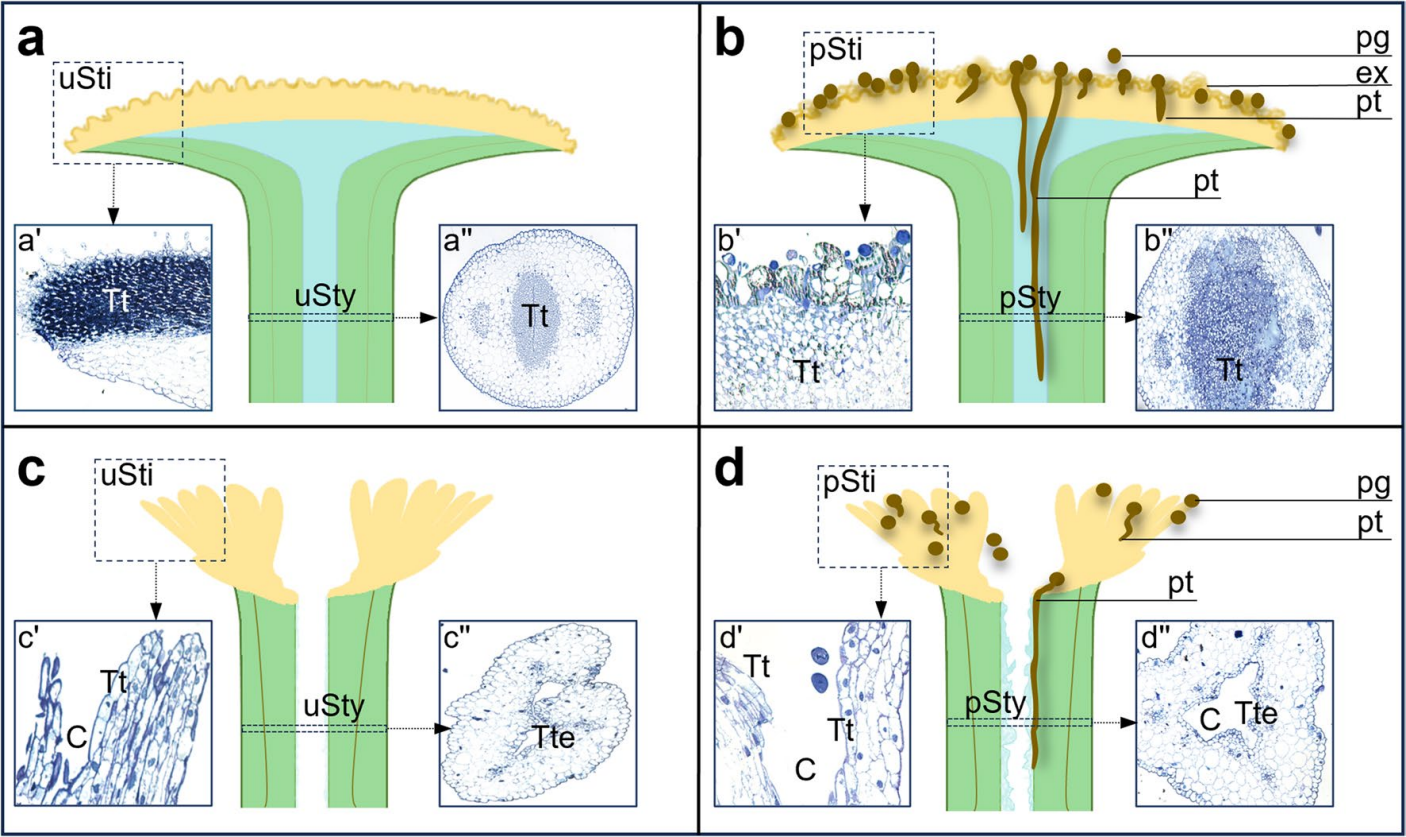
Graphical representation of *Petunia* and *Haemanthus* pistils. The cartoon illustrates the stigmas and styles of *Petunia* (**a**, **b**) and *Haemanthus* (**c**, **d**) before (**a**, **c**) and after pollination (**b**, **d**). Small photos depict methylene blue staining of stigmas ((a’)–(d’), longitudinal sections) and styles ((a’’)–(d’’), cross-sections). *C* canal, *ex* exudate, *pg* pollen grain, *pSti* pollinated stigma, *pSty* pollinated style, *pt* pollinated tube, *Tt* transmitting tissue, *Tte* transmitting tract epidermis, *uSti* unpollinated stigma, *uSty* unpollinated style

In this study, we presented a comprehensive immunogold localization of CRT within the nuclei of the pistil transmitting tract cells both before pollination and during the progamic phase. Our investigation encompasses two evolutionarily distant plant species, *Haemanthus albiflos* (monocots) and *Petunia hybrida* (dicots), which represent distinct anatomical types of stigmas and the pistil styles (Fig. 2). *Petunia* exhibits a wet stigma and solid style (Fig. 2a, b), while *Haemanthus* features a dry stigma and hollow style (Fig. 2c, d)*.* Through a comparative analysis of CRT’s nuclear localization pattern in these two plant species in relation to the exchangeable Ca^2+^, we explore the potential role of this protein within the cell nuclei of highly specialized somatic cells in the pistil transmitting tract.

## Materials and methods

### Plant material

Commercial cultivars of *Petunia hybrida* (angiosperms, dicots, Solanaceae family) and *Haemanthus albiflos* (angiosperms, monocots, Amaryllidaceae family) plants were grown at room temperature in the Department of Cellular and Molecular Biology, Faculty of Biological and Veterinary Sciences, Nicolaus Copernicus University in Toruń, Poland. Fresh pistils were gently dissected from flowers at two different developmental stages: (i) unpollinated pistil at anthesis and (ii) cross-pollinated pistil during the progamic phase, and fragments of receptive unpollinated/pollinated stigmas (u/pSti) and fragments of unpollinated/pollinated styles (u/pSty) were prepared according to standard protocols described below to obtain semi- and ultra-thin sections for light and electron microscopy. Plant material was derived from several growing seasons and the experiments were performed many times using many flowers in order to compare the obtained results, and representative data were shown.

### Histological and ultrastructural analyses

For histological and ultrastructural analyses, samples of u/pSti and u/pSty were fixed with freshly prepared 2.5% (v/v) glutaraldehyde (EM grade, Sigma-Aldrich/Merck) in phosphate-buffered saline (PBS, pH 7.2) for 2 h at room temperature (slight vacuum infiltration). After washing three times with PBS buffer, the samples were post-fixed in a 2% (v/v) aqueous solution of osmium tetroxide (OsO4_4_, EM grade, Polysciences), for 30 min at room temperature and rinsed twice with H_2_O mQ. Next, the fixed samples were dehydrated through a graded series of ethanol and embedded in Poly/Bed resin (Polysciences) according to the standard protocol. The embedded specimens were sectioned using a diamond knife (Micro Star Technologies) and a Leica UTC ultramicrotome. Semi-thin sections (longitudinal sections through the stigma and cross-sections through the style) were transferred onto microscope slides covered with Biobond (BioCell), stained with 0.1% methylene blue, and analyzed using a light microscope (Nikon Eclipse 80i). Ultra-thin sections (longitudinal or cross-sections through the stigma and style) were collected on copper grids, stained with 2.5% (w/v) uranyl acetate and 0.4% (w/v) lead citrate solutions, and examined by transmission electron microscopy (Jeol EM 1010) at 80 kV.

### Immunogold experiments

For immunogold labeling, samples were prepared as described previously (Lenartowski et al. 2015; Suwińska et. al. 2022) with several modifications. In brief, dissected samples of u/pSti and u/pSty were fixed with mixture of freshly prepared 4% (v/v) formaldehyde (EM grade, Polysciences) and 0.25% (v/v) glutaraldehyde (EM grade, Sigma-Aldrich/Merck) in PBS buffer (pH 7.2) for 1 h at room temperature (slight vacuum infiltration) followed by overnight fixation at 4 °C. Fixed samples were washed three times with PBS, dehydrated in graduated ethanol concentrations and embedded in LR Gold resin (Fluka) according to the standard protocol. The embedded specimens were sectioned as described above, and ultra-thin sections (longitudinal or cross-sections through the stigma and style) were collected on Formvar-coated nickel grids (Sigma-Aldrich/Merck). For reduction of non-specific antibody binding sites, the sections were incubated with blocking solution containing 3% (w/v) serum albumin (BSA, Sigma-Aldrich/Merck) in PBS buffer (pH 7.2), for 5 min at room temperature. In the next step, sections were incubated with the following primary antibodies: (i) a rabbit polyclonal antibody against maize CRT (CRT PAb, Napier et al. 1995), (ii) a rabbit polyclonal antibody against CRT PAb (Proteintech), and (iii) a rabbit polyclonal antibody against CNX1/2 PAb (CNX1/2 homolog from *Arabidopsis thaliana*, Agrisera). The primary antibodies were diluted in PBS buffer (dilution 1:20 for non-commercial CRT PAb and 1:50 for commercial CRT and CNX1/2 PAbs) supplemented with 0.3% (w/v) BSA (for CRT immunostaining) or 0.5% (w/v) BSA (for CNX1/2 immunostaining). Ultra-thin sections were incubated with the primary antibodies, for 2 h at room temperature, followed by incubation with a gold-conjugated (15 nm) goat anti-rabbit IgG antibody (BBInternational), diluted 1:100 in PBS buffer with 0.2% (w/v) BSA (for CRT immunostaining) or 0.5% (w/v) BSA (for CNX1/2 immunostaining), for 1.5 h at room temperature. Sections were washed several times in PBS buffer between incubations with the primary and secondary antibodies. In the control reaction, incubation with the primary antibody was omitted. Finally, the sections were rinsed twice with H_2_O mQ, stained with 2.5% (w/v) solution, and examined by transmission electron microscopy as above. Although the specificity of maize CRT PAb in *Petunia* and *Haemanthus* tissues was previously verified by immunoblotting (Lenartowska et al. 2009; Lenartowski et al. 2015), we performed Western blot analysis for all primary antibodies used.

The quantitative and statistical analyses were collectively performed for approximately 90 cross-sections of nuclei. Results were expressed as the number of gold particles per nuclei cross-section derived from each species (*Petunia*, *Haemanthus*) ± SD (standard deviation). Data were analyzed using a non-parametric one-tail Mann–Whitney test. Statistical significance was defined as follows: ns not significant, *p < 0.05, **p < 0.01, ***p < 0.001 (to compare the number of gold traces before and after pollination).

### Potassium antimonate precipitation

Localization of exchangeable (loosely bound) Ca^2+^ was performed according to the protocol described previously (Lenartowski et al. 2015; Suwińska et al. 2022). Briefly, dissected samples of u/pSti and u/pSty were fixed with freshly prepared 2% (w/v) potassium antimonate (Sigma-Aldrich/Merck), 2% (v/v) glutaraldehyde (EM grade, Sigma-Aldrich/Merck), and 2% (v/v) formaldehyde (EM grade, Polysciences) in 0.1 M phosphate buffer (KH_2_PO_4_, pH 7.8) for 4 h at room temperature. Next, the samples were washed several times in the phosphate buffer and post-fixed with 1% (v/v) OsO_4_ (EM grade, Polysciences) in the same buffer-antimonate solution for 12 h at 4 °C. After washing with H_2_O mQ, the samples were dehydrated in graduated ethanol concentrations, and embedded in Spurr resin (Merck) according to the standard protocol. The embedded specimens were sectioned as described above. Ultra-thin sections (longitudinal or cross-sections through the stigma and style) were collected on copper grids, stained with 2.5% (w/v) uranyl acetate and 0.4% (w/v) lead citrate solutions, and examined by transmission electron microscopy as above. The presence of Ca^2+^ in the Ca^2+^-antimonate precipitates (Ca^2+^ ppts) was confirmed previously using energy-dispersive X-ray microanalysis (Bednarska et al. 2005).

### Western blotting

Immunoblotting was performed to verify the specificity of the primary antibodies used according to the previously described protocol (Lenartowski et al. 2015). Briefly, 100 mg of tissues (*Petunia*, *Haemanthus*, *Arabidopsis*, and maize pistils, and mouse testis) were homogenized in liquid nitrogen. A soluble fraction of proteins was extracted by 50 mM HEPES (pH 7.5), 10% sucrose, 5 mM EGTA, 5 mM EDTA, 2 mM DTT, and cOmplete™ Protease Inhibitor Cocktail (Roche) according to the manufacturer’s recommendation. Homogenates were centrifuged at 16,000 g, 30 min, 4 °C. Protein concentration was spectrophotometrically quantified (Bio-Rad DC Protein Assay), and equal amounts of proteins (20 µg of proteins per well) were separated on a 10% SDS-PAGE gels. Next, proteins were semi-dry transferred to Immun-Blot LF PVDF (Bio-Rad), and blocked blots were probed with maize CRT PAb produced by Napier et al. (1995), commercial human CRT PAb (27298–1-AP, Proteintech), and *Arabidopsis* CNX1/2 PAb (AS122365, Agrisera) with following dilutions 1:5000, 1:1500, and 1:5000, respectively. After that, the membranes were washed and probed with the anti-rabbit IgG antibody conjugated with horseradish peroxidase (HRP, Merck), and signal was visualized using the Amersham ECL Advance Western Blotting Detection Kit according to the manufacturer’s instructions (GE Healthcare).

## Results

### Nuclear-specific localization of CRT in relation to exchangeable Ca^2+^ in *Petunia* pistil transmitting tract cells before and after pollination

**Fig. 3:**
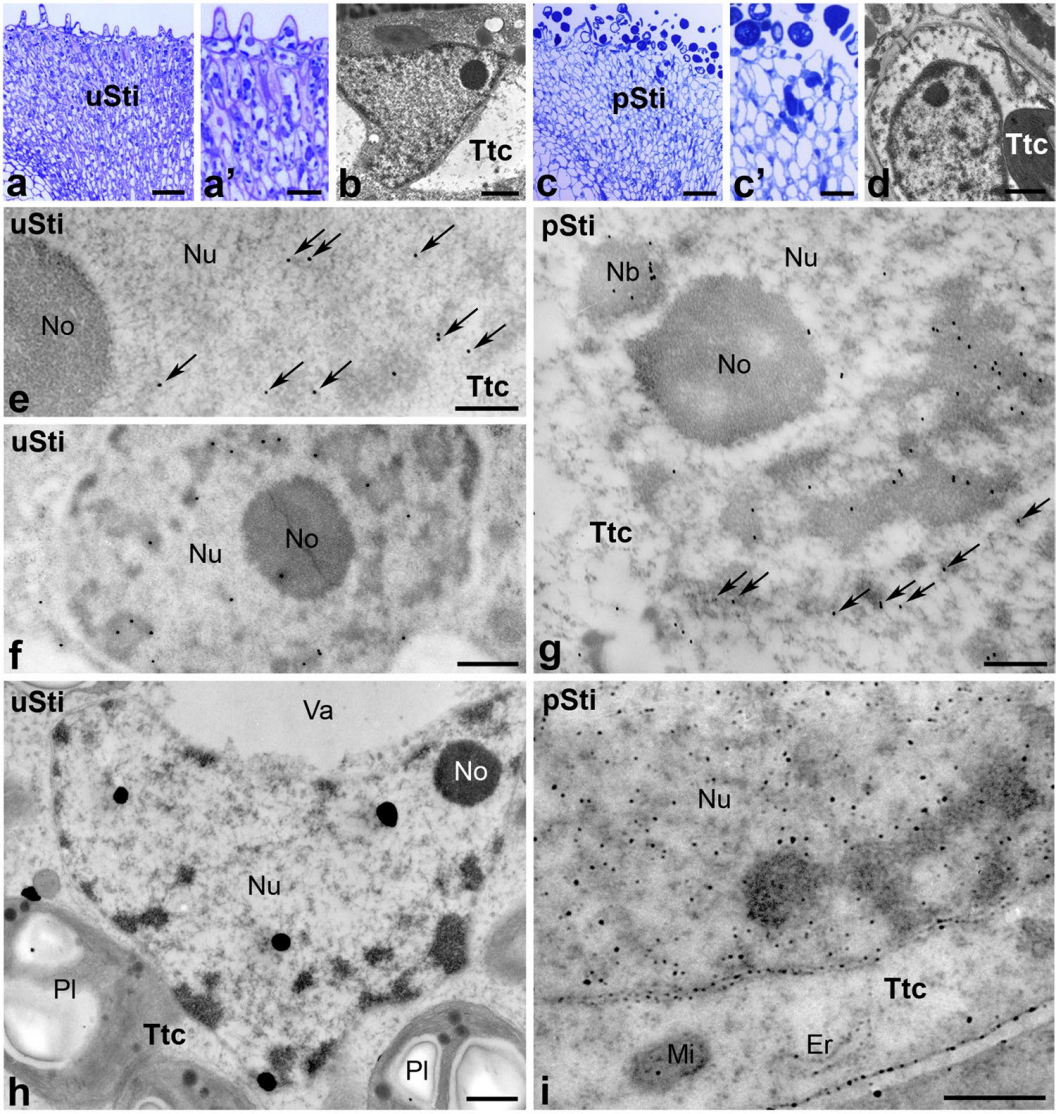
Immunogold distribution of CRT (**e**–**g**) and visualization of loosely bound Ca^2+^ (**h**, **i**) in *Petunia* stigmas before and after pollination. Methylene blue-stained longitudinal sections of the uSti (**a**, (a’)) and pSti (**c**, (c’)). Ultrastructure of transmitting tract cell (*Ttc*) before (**b**) and after pollination (**d**). Localization of CRT in uSti (**e**, **f**) and pSti (**g**). Distribution of exchangeable Ca.^2+^ in uSti and pSti (**h** and **i**, respectively). *Er* endoplasmic reticulum, *Mi* mitochondria, *Nb* nuclear bodies, *No* nucleolus, *Nu* nucleus, *Pl* plastids, *Ttc* transmitting tissue cells, *Va* vacuole. *Bars* 50 µm (**a**, **c**), 25 µm ((a’), (c’)), 1 µm (**b**, **d**, **h**, **i**), 200 nm (**e**–**g**)

**Fig. 4:**
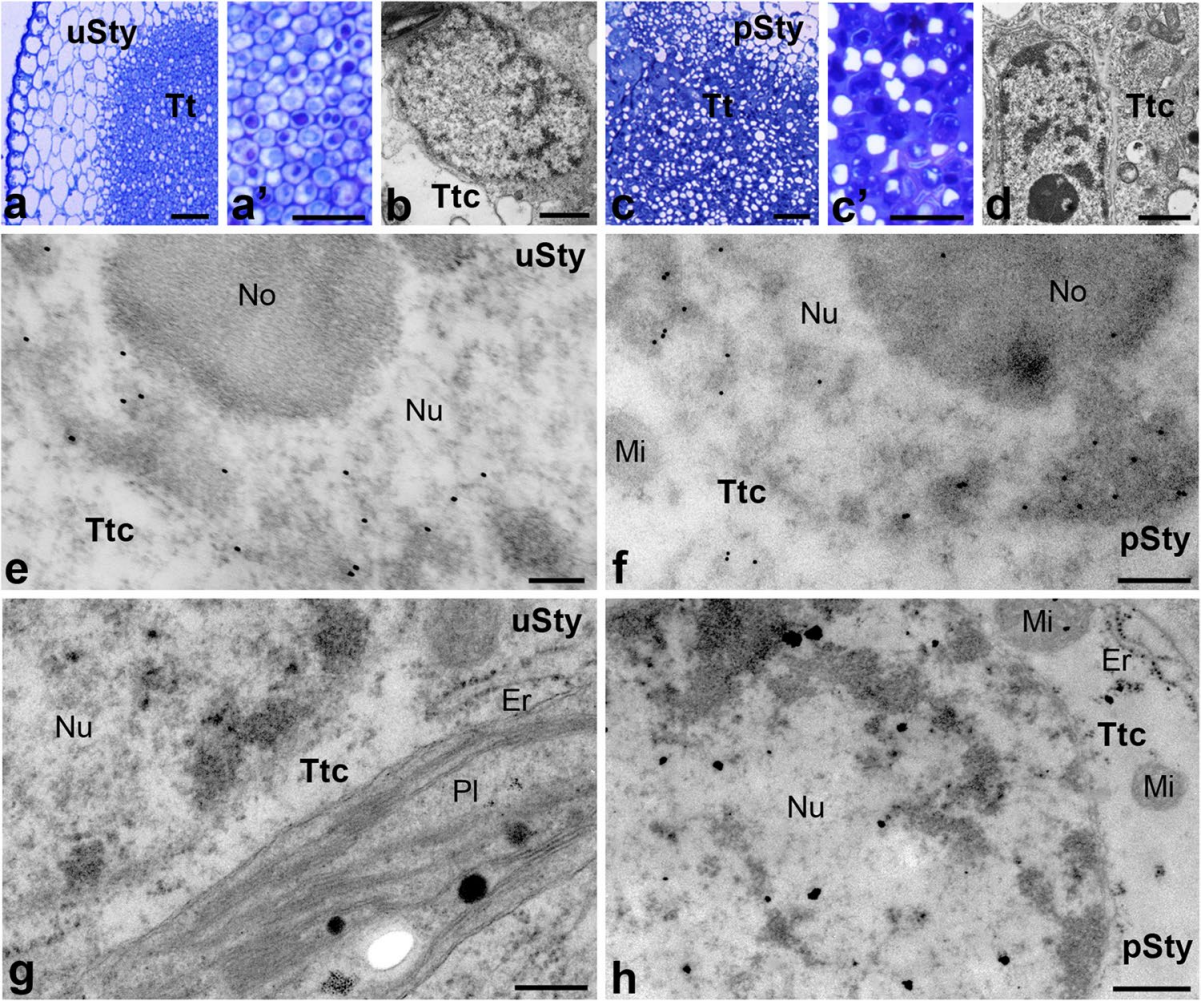
Immunogold distribution of CRT (e–f) and visualization of loosely bound Ca^2+^ (**g**, **h**) in *Petunia* styles before and after pollination. Methylene blue-stained cross-sections of the uSty (**a**, (a’)) and pSty (**c**, (c’)). Ultrastructure of transmitting tract cell (*Ttc*) before (**b**) and after pollination (**d**). Localization of CRT in uSty (**e**) and pSty (**f**). Distribution of exchangeable Ca.^2+^ in uSty and pSty (**g** and **h**, respectively). *Er* endoplasmic reticulum, *Mi* mitochondria, *No* nucleolus, *Nu* nucleus, *Pl* plastids, *Tt* transmitting tissue, *Ttc* transmitting tissue cells. *Bars* 25 µm (**a**, (a’), **c**, (c’)), 1 µm (**b**, **d**), 200 nm (**e**–**h**)

The *Petunia* pistil consists of a wet stigma covered with exudate at the receptive stage (Fig. 2a(a’)) and a solid style filled with specialized transmitting tissue forming a central core (Fig. 2a(a”)). Methylene blue-stained semi-thin sections reveal that the surface of *Petunia* wet stigma is relatively smooth with numerous unicellular stigmatic papillae (Figs. 2(a’), 3a(a’)). The transmitting tract, connecting the stigma with the ovary in *Petunia*, (both the stigma transmitting tract and the transmitting tissue of the style), comprises highly secretory cells responsible for producing an extensive extracellular matrix to provide nutrition, adhesion, and guidance for pollen tube growth. These small and compact cells (Figs. 3a(a’), 4a(a’)) are elongated along the stigma-ovary axis (Fig. 3(a’)). After pollination, numerous pollen grains germinate on the stigma (Figs. 2b(b’), 3c(c’)) and pollen tubes elongate into the style (Figs. 2b(b”), 4c(c’)). During the progamic phase, both the exudate covering the stigma surface and the extracellular matrix of the pistil transmitting tract increase rapidly (Figs. 2b(b’), (b”), 4c(c’)). Ultrastructural analysis using transmission electron microscopy reveals that all cells forming the transmitting tract in *Petunia* have large, active cell nuclei with nucleoli located in their center. Areas of condensed chromatin are mainly located not only at the periphery of the cell nuclei, but also in their center (Figs. 3b, d, 4b, d). Nuclear micro-images are similar both before (Figs. 3b, 4b) and after pollination (Figs. 3d, 4d).

In our initial analysis of CRT and Ca^2+^ ppts localization in the transmitting tract cells of the u/pSti, we observed CRT PAb signal inside the nucleus before pollination. These signals were primarily associated with perichromatin areas (Fig. 3e, *arrows*, 3f). Additionally, immunolabeling was found in dense chromatin and interchromatin regions (Fig. 3e, f), and only single gold traces were found in the nucleolus (Fig. 3f). To investigate whether pollination, along with pollen germination and pollen tube growth in the stigma transmitting tract, induces changes in the level or pattern of CRT localization in nuclei, we analyzed the pSti electron micrographs. As shown in Fig. 3g, we revealed a similar pattern of the CRT PAb labeling in the nuclei of pollinated stigma. Furthermore, CRT PAb signals were also detected in nuclear bodies (Fig. 3g), and numerous gold traces were localized on the periphery of the nucleus, possibly corresponding to the nuclear envelope or the ER adjacent to the nuclear envelope (Fig. 3g, *arrows*).

It is essential to note that Ca^2+^ in cells exists in several states: (i) cytosolic free Ca^2+^ (ionic form) is freely soluble and acts as a second messenger in cell signaling; (ii) insoluble covalently bound Ca^2+^ plays mainly a structural role, particularly in such dense crystalline forms as calcium crystals and cell walls; (iii) loosely bound Ca^2+^ is the prevalent form of Ca^2+^ stored in most cells and often sequestered in the cell wall or localized in specific organelles, such as ER (Ge et al. 2007). Stored Ca^2+^ is typically associated with fixed/mobile anions or proteins that control Ca^2+^ concentration. This pool of Ca^2+^ is exchangeable and can transform into other forms when and where it is needed and can be detected by competition with low-affinity anions, such as antimonate. Because exchangeable Ca^2+^ can be associated with Ca^2+^-binding/buffering proteins that control Ca^2+^ homeostasis, we hypothesized that CRT localization sites and exchangeable Ca^2+^ may correlate in the nuclei of the stigma transmitting tract cells. To verify this hypothesis, we visualized loosely bound Ca^2+^ using the potassium antimonate precipitation method in *Petunia* u/pSti. As expected, only single electron-dense Ca^2+^ ppts were found associated with the nuclear chromatin of the uSti transmitting tract, mainly in perichromatin areas (Fig. 3h). Compared to uSti, a significantly higher level of exchangeable Ca^2+^ was observed after pollination with numerous Ca^2+^ ppts localized in chromatin and associated with the nuclear envelope/ER (Fig. 3i). Furthermore, Ca^2+^ ppts were also detected in some other cellular compartments, such as the ER, mitochondria, and the cell wall (Fig. 3i).

Next, we analyzed the nuclear localization of CRT and Ca^2+^ ppts in the transmitting tissue that forms a central core in the solid style of *Petunia*. This highly specialized transmitting tissue connects the stigma and ovary and is composed of secretory cells with very large cell nuclei (Fig. 4b, d). In the transmitting tissue cells of both uSty and pSty, CRT was primarily detected in perichromatin areas (Fig. 4e, f). However, after pollination, the labeling was also in the nucleolus-associated chromatin, and few gold particles were observed inside the nucleolus (Fig. 4f). In both *Petunia* uSty and pSty, Ca^2+^ ppts in the transmitting tissue cell nuclei were predominantly localized in perichromatin areas. However, their level increased after pollination (Fig. 4g, h). Additionally, single electron-dense Ca^2+^ ppts of various sizes were also detected in plastids (Fig. 4g), ER, and mitochondria (Fig. 4h).

**Fig. 5:**
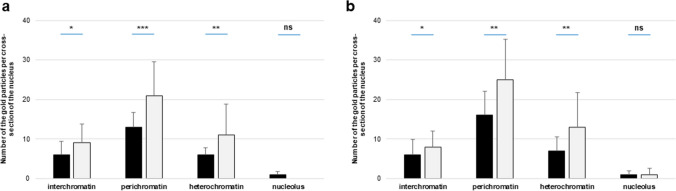
Statistical analysis of the gold particle distribution within nuclear compartments of *Petunia* stigmas (**a**) and styles (**b**). Graphs show number of gold particles within analyzed nuclear compartments. The dark and light bars represent stages before and after pollination process, respectively. Statistical significance was carried out by Mann–Whitney test, ns not significant, *p < 0.05, **p < 0.01, ***p < 0.001

The determination of CRT localization within the cell nucleus raised the question of whether the pollination process induces changes in the level of this protein in this cellular compartment. To address this, we analyzed electron microscopy images for the quantity of visible gold particles, with particular attention to defined nuclear subdomains. Our observations were further supported by statistical analysis (Fig. 5). In the stigma, we detected a significant increase in CRT PAb labeling after pollination, mainly in the perichromatin and heterochromatin regions, which amounted to 62% and 83%, respectively. Additionally, we noted a lower increase in CRT level in the interchromatin regions of pSti (50%) (Fig. 5a). We obtained a similar relationship for the style of *Petunia*, where the greatest increase in the quantity of gold particles after pollination occurred in heterochromatin (86%), and to a lesser extent in the peri- and interchromatin areas (56% and 33%, respectively) (Fig. 5b).

### Nuclear-specific localization of CRT in relation to exchangeable Ca^2+^ in Haemanthus pistil transmitting tract cells before and after pollination

**Fig. 6:**
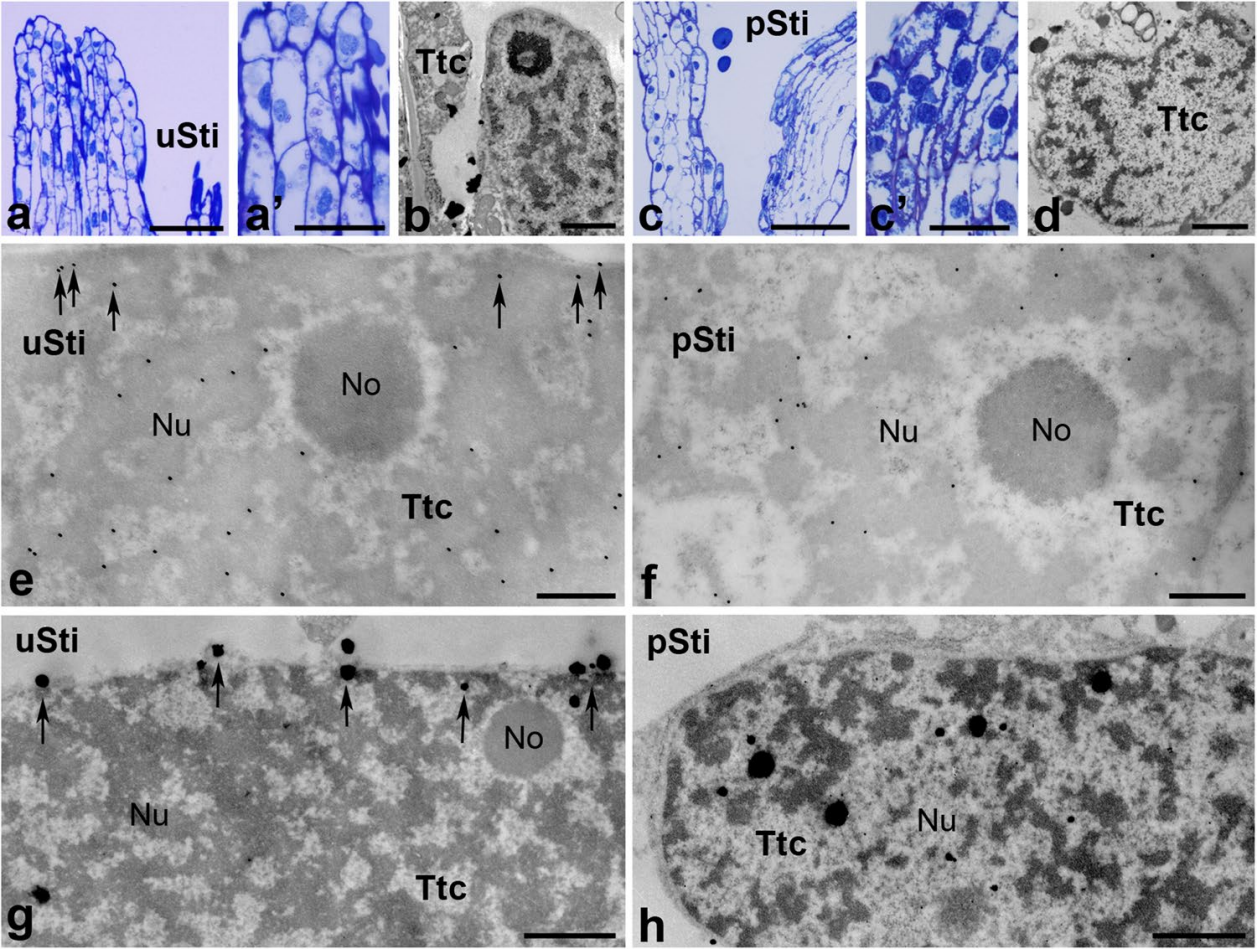
Immunogold distribution of CRT (**e**, **f**) and visualization of loosely bound Ca^2+^ (**g**, **h**) in *Haemanthus* stigmas before and after pollination. Methylene blue-stained longitudinal sections of the uSti (**a**, (a’)) and pSti (**c**, (c’)). Ultrastructure of transmitting tract cell (*Ttc*) before (**b**) and after pollination (**d**). Localization of CRT in uSti (**e**) and pSti (**f**). Distribution of exchangeable Ca.^2+^ in uSti and pSti (**g** and **h**, respectively). *No* nucleolus, *Nu* nucleus, *Ttc* transmitting tissue cells. *Bars* 50 µm (**a**, **c**), 25 µm ((a’), (c’)), 1 µm (**b**, **d**, **g**, **h**), 500 nm (**e**, **f**)

**Fig. 7:**
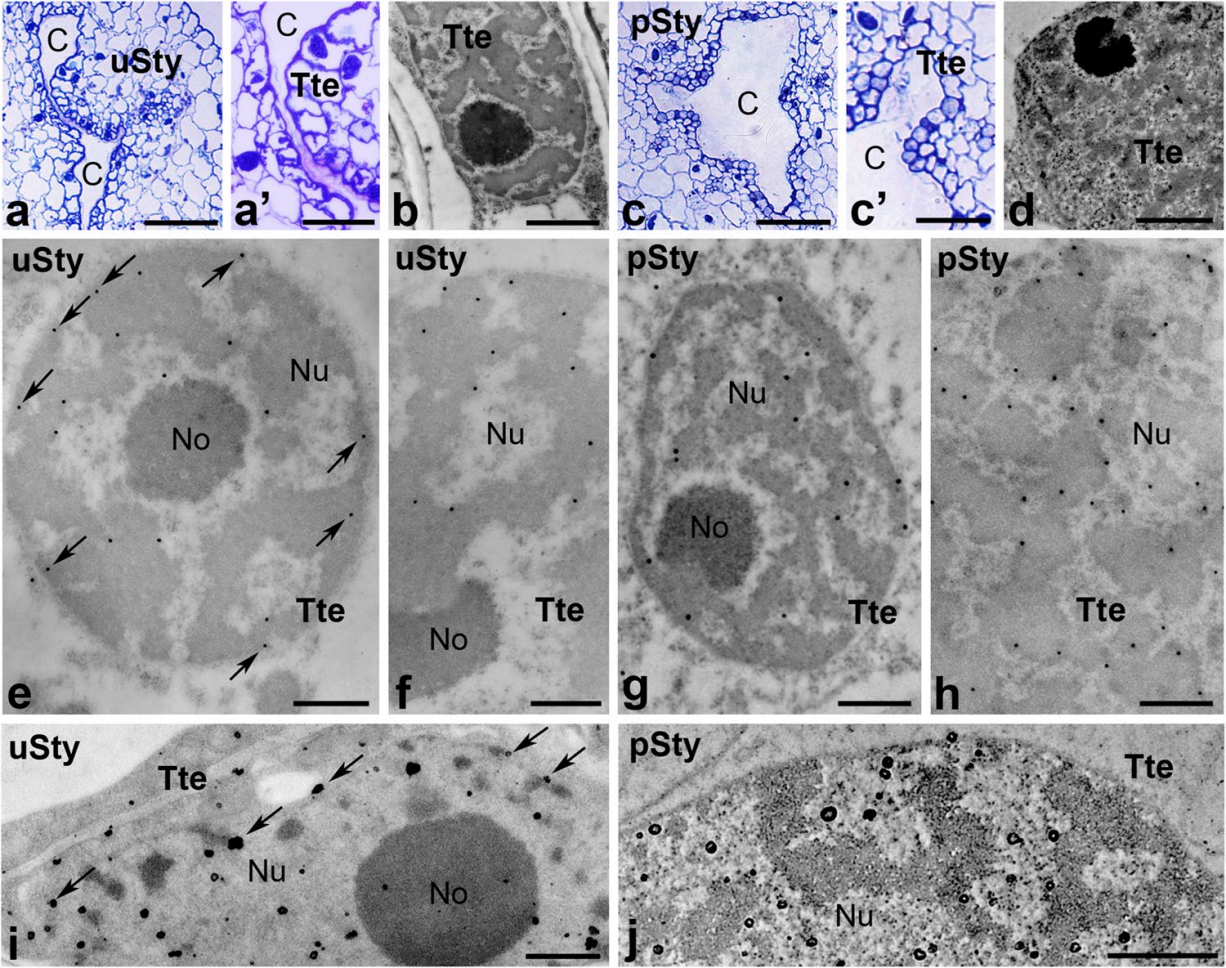
Immunogold distribution of CRT (**e**–**h**) and visualization of loosely bound Ca^2+^ (**i**, **j**) in *Haemanthus* styles before and after pollination. Methylene blue-stained cross-sections of the uSty (**a**, (a’)) and pSty (**c**, (c’)). Ultrastructure of transmitting tract cell (*Ttc*) before (**b**) and after pollination (**d**). Localization of CRT in uSty (**e**, **f**) and pSty (**g**, **h**). Distribution of exchangeable Ca.^2+^ in uSty and pSty (**i** and **j**, respectively). *C* canal, *No* nucleolus, *Nu* nucleus, *Tte* transmitting tissue epidermis. *Bars* 50 µm (**a**, **c**), 25 µm ((a’), (c’)), 1 µm (**b**, **d**, **i**, **j**), 500 nm (**e**, **h**)

*Haemanthus* is characterized by a dry stigma and a hollow style, in contrast to *Petunia*. Compared to other plant species with dry stigmas, *Haemanthus* has a typical papillate stigma without exudate on the surface, even in the receptive phase (Figs. 2c(c’), 6a(a’)) and after pollination (Figs. 2d(d’), 6c(c’)). The style is hollow (Figs. 2c, d, 7a, c), with the stylar canal limited by a layer of inner epidermis cells, as shown in semi-thin cross-sections stained with methylene blue (Figs. 2(c”), (d”), 7(a’), (c’)). During the progamic phase, a small amount of secretion resulting from the secretory activity of the inner epidermis fills the stylar canal (Fig. 2d). Pollen grains that reached the stigmatic papillae germinate (Figs. 2d, 6c), and the pollen tubes grow into the style towards the ovary at the surface of the inner epidermis (Fig. 2d). The pistil transmitting tract in *Haemanthus* (stigma papillae and stylar inner epidermis) consists of compact cells elongated along the stigma-ovary axis (Fig. 6(a’), (c’)). They are responsible for adhesion, nutrition, and guidance of elongating pollen tubes. As shown in semi-thin sections stained with methylene blue (Figs. 6(c’), 7(a’)) as well as in ultra-thin sections (Figs. 6b, d, 7b, d), transmitting tract cells have large nuclei with relatively large areas of dense chromatin and active nucleoli. Ultrastructural analysis using transmission electron microscopy showed that nuclear micro-images were similar both before (Figs. 6b, 7b) and after pollination (Figs. 6d, 7d).

Once again, we analyzed the distribution of CRT and Ca^2+^ ppts in the nuclei of *Haemanthus* transmitting tract cells before and after pollination. For this purpose, we initially investigated the localization of the gold traces corresponding to the CRT PAb in u/pSti and revealed that CRT is mainly localized with nuclear chromatin (Fig. 6e, f). The labeling was associated with perichromatin areas as well as with dense chromatin. However, before pollination, some gold tracers were specifically detected at the peripheral region of the nucleus (Fig. 6e, *arrows*). One of the most interesting observations was that in uSti, most Ca^2+^ ppts accumulated at the border of the nucleus and cytoplasm (Fig. 6g, *arrows*), whereas in pSti, they were evenly distributed in the nucleoplasm (Fig. 6h).

While the wet stigma in *Petunia* is connected to the transmitting tissue of the solid style, the dry stigma of *Haemanthus* passes into the stylar canal of the hollow style (Fig. 2). As previously, we analyzed the patterns of CRT and Ca^2+^ ppts localization in the nuclei of the inner epidermal cells, as these cells correspond to the *Petunia* transmitting tissue. Before pollination, we observed gold traces corresponding to CRT in chromatin, mainly in perichromatin areas (Fig. 7e). However, the gold particles were often localized peripherally (Fig. 7e, *arrows*) and much less frequently in dense chromatin (Fig. 7f). After pollination, we did not observe any significant changes in the level or pattern of nuclear CRT labeling in *Haemanthus* stylar transmitting tract cells (compare Fig. 7e and g). Finally, we compared the localization patterns of Ca^2+^ ppts in the nuclei of the inner epidermal cells. We found that exchangeable Ca^2+^ was common in the chromatin both before and after pollination. However, in uSty, numerous Ca^2+^ppts were localized peripherally, at the border of the nucleus and cytoplasm (Fig. 7i, *arrows*), whereas in pSty, most of the Ca^2+^ ppts were associated with the perichromatin areas (Fig. 7j). It should be emphasized that the Ca^2+^ ppts localization pattern corresponded to the CRT localization pattern both before and after pollination.

**Fig. 8:**
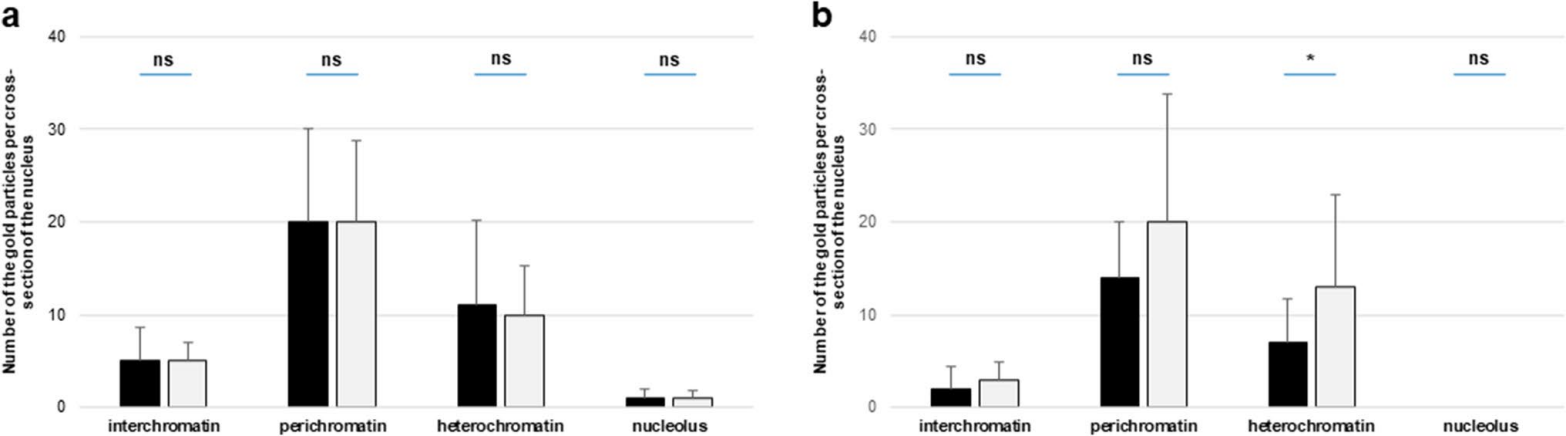
Statistical analysis of the gold particle distribution within nuclear compartments of *Haemanthus* stigmas (**a**) and styles (**b**). Graphs show number of gold particles within analyzed nuclear compartments. The dark and light bars represent stages before and after pollination process, respectively. Statistical significance was carried out by Mann–Whitney test, ns not significant, *p < 0.05, **p < 0.01, ***p < 0.001

Similarly to *Petunia*, we compared the distribution level of CRT before and after pollination for *Haemanthus* (Fig. 8). It should be noted that for the stigma, we did not observe significant differences in the level of CRT labeling resulting from the pollination process (Fig. 8a). On the contrary, a statistically significant increase in the level of gold particles, amounting to 86%, was identified in the heterochromatin region of the style. For the other compartments, we did not observe any differences, or they were statistically insignificant (Fig. 8b).

### Control experiments

To reinforce our findings, we conducted a series of control experiments based on electron microscopy and immunoblotting techniques. First, the negative control, omitting the primary CRT PAb, showed no labeling within the nuclei of transmission tract cells of both *Petunia* (Fig. S1a) and *Haemanthus* (Fig. S1b) species. The arrow points to sporadically appearing individual traces of gold in the nucleus of *Petunia* (Fig. S1a). Moreover, to confirm the specificity of the primary antibody against maize CRT, a Western blot experiment was performed (Fig. S1c) and revealed the presence of a single band corresponding to CRT in the tested plant species.

Second, although the maize CRT PAb (Napier et al. 1995) used in this work is highly specific for plant CRT what we have shown in our previous studies (Lenartowska et al. 2009; Lenartowski et al. 2015; Niedojadło et al. 2015; Suwińska et al. 2015; 2017; 2022), we wanted to test whether the use of another antibody against CRT would confirm the presence of this protein in the nuclei of plant cells. For this purpose, we used a commercial CRT PAb (Proteintech) during immunolabeling experiments (Fig. S2). This control reaction confirmed our results and showed that in both, uSty and pSty of *Petunia* and *Haemanthus* the CRT PAb labeling was found in cell nuclei of transmitting tract cells (Fig. S2a, b, d, e). In both species, the negative controls were generally unlabeled, with only single gold traces in the nuclei (Fig. S2c, f). In addition, Western blot analysis confirmed the specificity of the commercial CRT PAb in plant protein extracts (Fig. S2g).

Finally, we determined the distribution of calnexin (CNX) in transmitting tract cells of *Petunia* uSty/pSty. CNX and CRT share a common evolutionary origin and arose from the duplication of a single ancestral gene (Del Bem 2011). Moreover, both proteins are components of the CNX/CRT cycle, which is responsible for monitoring the correct folding and removal of improperly folded proteins before they exit the ER. However, CNX is an ER-transmembrane protein, which limits its mobility, whereas CRT is a soluble ER luminal protein, and its presence was confirmed also outside of the ER (Michalak 2023). Therefore, only CRT can exist in the cell nucleus. In fact, using a commercial CNX1/2 PAb, we confirmed the presence of CNX in the ER membranes of *Petunia* u/pSty transmitting tract cells, while this protein was absent in the nuclei of these cells (Fig. S3a, b). A negative control was also performed and showed no immunogold labeling (Fig. S3c). At last, the Western blotting experiment confirmed the specificity of CNX1/2 PAb in *Petunia* and *Haemanthus* protein extracts (Fig. S3d).

## Discussion

In this study, we present a detailed analysis of the immunogold localization of CRT in relation to exchangeable Ca^2+^ in the nuclei of highly specialized somatic cells of the pistil transmitting tract cells before and after pollination. Similar patterns of nuclear distribution of CRT and loosely bound Ca^2+^ were observed in two different plant species, *Petunia* and *Haemanthus*, which differ in the anatomical structure of the pistil. The primary localization sites of CRT and Ca^2+^ included perichromatin areas, dense chromatin, and the periphery of the cell nucleus with the nuclear envelope. However, following pollination, additional CRT localization sites were identified, such as nuclear bodies, nucleolus, and nucleolus-associated chromatin. Furthermore, there was an observed increase in the level of CRT labeling in *Petunia* and an increase in exchangeable Ca^2+^ levels in both plant species after pollination. This suggests dynamic changes in the distribution and levels of CRT and Ca^2+^ in response to the pollination process.

The domain structure of CRT is characterized by the presence of an N-terminal ER-targeting signal and a C-terminal ER-retention signal (mostly HDEL in plants and KDEL in animals) (Jia et al. 2009; Michalak 2023). Our phylogenetic analysis of plant CRT homologs confirmed that all identified plant CRTs contain these conserved signal sequences, emphasizing the ER lumen as the primary site of localization and function for these proteins (Wasąg et al. 2019). Previous experimental work has provided molecular evidence supporting the translation of CRT on the rough ER of cultivated pollen tubes (Suwińska et al. 2015). Additionally, CRT has been shown to be highly expressed and accumulated in the ER during pollen development, pollen tube growth in vitro, and pollen-pistil interactions (Lenartowska et al. 2009; Lenartowski et al. 2014, 2015; Wasąg et al. 2018; Suwińska et al. 2022). Despite the expected ER localization, experimental findings from various authors have revealed the existence of plant CRT in other cellular compartments, including dictyosomes, cytoplasm, nucleus, plasma membrane, and even the extracellular matrix (as indicated in several publications in the introduction). While the localization and function of plant CRT outside the ER are commonly accepted, the mechanisms enabling the movement of this protein between different cellular compartments remain unknown. It has been speculated that more than one pool of CRT exists in eukaryotic cells, including a special nuclear-specific isoform (Jethmalani et al. 1997; Roderick et al. 1997). Presently, at least three CRT isoforms in plant genomes are characterized, differing slightly in structure and function, especially in relation to the CRT1/2 and CRT3 subclasses (Persson et al. 2003; Wasąg et al. 2019). Notably, each isoform contains both the signal sequence and HDEL retention motif, suggesting that alternative hypotheses explaining the nuclear localization of CRT should be explored. One of the hypotheses proposes that proteolytic degradation of the C-domain containing the ER-retention sequence may result in the release of CRT outside the ER (Gold et al. 2010). However, in the absence of experimental evidence supporting this hypothesis, it remains speculative. Another possibility suggests direct protein–protein interactions or enzymatic modifications as factors causing conformational changes in CRT structure, leading to the redistribution of CRT outside the ER (Jethmalani et al. 1997; Corbett et al. 2000; Navazio et al. 2002; Persson et al. 2003; Afshar et al. 2005; Shaffer et al. 2005; Decca et al. 2007; Carpio et al. 2010; Goitea and Hallak 2015). It is postulated that these biochemical processes may be triggered by local/transient changes in cellular conditions, such as pH, ROS, ATP, and ion (Ca^2+^, Zn^2+^, Mg^2+^) concentrations (Corbett et al. 2000; Carpio et al. 2010; Gold et al. 2010). For example, proteolytic cleavage results in the appearance of an additional population of cytosolic CRT derived from the ER luminal fraction, as confirmed for the soluble pool of rat brain proteins and in vitro experiments (Afshar et al. 2005; Shaffer et al. 2005; Decca et al. 2007). The binding of Ca^2+^, Zn^2+^, and Mg^2+^-ATP induces conformational changes in CRT’s tertiary organization, providing a more compact and protease-resistant structure for the protein (Corbett et al. 2000). Simultaneously, a decrease in Ca^2+^ promotes the proteolysis of the N-domain (containing the signal peptide) and the appearance of an arginylation site. This process takes place in the cytosol after retranslocation of the truncated protein molecule (Afshar et al. 2005; Decca et al. 2007; Carpio et al. 2010). Notably, arginylation of CRT appears to be an inefficient substrate for the proteasomal degradation, resulting in a longer half-life compared to non-modified CRT (Afshar et al. 2005; Goitea and Hallak 2015). Moreover, post-translational arginylation directs the protein to structures called stress granules (Decca et al. 2007). These results suggest that the localization of CRT outside the ER may be the result of changes in the cell microenvironment in both physiological or pathological states.

This study focusses on the localization and probable function of CRT in the nuclei of transmitting tract cells of unpollinated and pollinated pistils. It is noteworthy that the presence of this protein in different nuclear domains, including chromatin, nuclear matrix, and the nuclear envelope of both animal and plant cells, has been previously reported (Opas et al. 1991; Dedhar et al. 1994; Denecke et al. 1995; Napier et al. 1995; Roderick et al. 1997; Brünagel et al. 2003; Jia et al. 2008; Popłońska 2013; Iborra and Papadopoulos 2017). Previous results of our team also indicated the possibility of nuclear CRT localization in different *Petunia* cells (Lenartowski et al. 2015; Suwińska et al. 2022). The nuclear localization of CRT is supported by mapping of a putative NLS in animal and plant species (Michalak et al. 1992; Denecke et al. 1995; Borisjuk et al. 1998; Mushtaq et al. 2020). The NLS (PPKXIKDPX) appears to be evolutionarily conserved and located within the P-domain of CRT, partially overlapping with repeat 1 of the characteristic triplicate motif M1 (Michalak et al. 1992; Lenartowski et al. 2014; Wasąg et al. 2022). Our bioinformatics analysis confirmed the presence of the putative NLS sequence in CRTs of all examined plants to date (Wasąg et al. 2019), with the degree of identity of the NLS motif ranging from 57 to 88% (data not shown). Although the concept of the presence of an NLS motif in the CRT sequence seems probable, it should be noted that its functionality has not been experimentally confirmed so far, but has only been identified based on in silico analysis. In this study, we confirmed the presence of CRT within the nucleus and nuclear envelope before and after pollination in *Petunia*. There was an increased level of the protein observed after pollination in both the pistil stigma and style. Additionally, we identified novel localizations of the protein in the pollinated pistil, such as nuclear bodies and an increased accumulation of gold particles within dense chromatin and nuclear periphery areas (stigma), as well as in chromatin associated with the nucleolus (style). Similar observations were made with Ca^2+^, which increased after pollination within the nuclei of the pistil transmitting tract, particularly significant in the nuclear envelope. Regarding *Haemanthus*, CRT and Ca^2+^ localization patterns within transmission tract cell nuclei were generally similar. CRT predominantly occupied perichromatin and dense chromatin regions (stigma), with some gold traces preferentially occurred at the peripheral region of the nucleus (style). Localization of exchangeable Ca^2+^ was mainly limited to the border of the nucleus and cytoplasm of the transmission tissue (unpollinated stigma and style) and nucleoplasm/perichromatin areas (pollinated stigma and style). Despite this, significant differences in the levels of CRT and Ca^2+^ labeling before and after pollination were not detected. We are aware that the optimal methodological approach would involve simultaneous visualization of CRT and Ca^2+^ to capture their mutual correlation. However, the fundamental disparity between both reactions—immunocytochemical for CRT and cytochemical for Ca^2+^—precludes this possibility, both in terms of cellular imaging (due to the markedly different preparation of biological material) and statistical analyses. The immunogold reaction signal, manifested as colloidal gold grains of uniform size, allows for precise statistical analysis of labeling levels. In contrast, in the case of potassium antimonate precipitation, Ca^2+^ ppts of very different sizes are formed, which may lead to significant errors in the interpretation of the obtained results of statistical analyses. Therefore, in our research, we have proposed a subjective assessment of the Ca^2+^ level in relation to CRT, mirroring the approach taken in our previous publications (Lenartowska et al. 2009; Lenartowski et al. 2015; Wasąg et al. 2018; Suwińska et al. 2022).

The sub-nuclear localization pattern of CRT highlights the diversity of its functions and involvement in various cellular processes. As currently known, the interchromatin comprises numerous sub-compartments collectively described as nuclear bodies (Shan et al. 2023). Moreover, the interchromatin space is associated with specific types of proteins and regulatory RNAs that form functional complexes involved in transcription, splicing, replication, and repair processes occurring directly within the interchromatin or in the perichromatin region (Cremer et al. 2020). It was shown that colocalization of the CRT with nuclear bodies, such as Cajal bodies, suggests its participation, at least, in pre-mRNA splicing machinery (Shaw and Brown 2004). Similarly, CRT’s association with the perichromatin compartment of the nucleus indicates its potential role in transcription and post-transcriptional processing (Biggiogera et al. 2008). Generally, it is suggested that at the interface of these two subdomains, most DNA-related processes occur during interphase (Masiello et al. 2018; Cremer et al. 2020; Shan et al. 2023). Moreover, CRT may play a role in *Chara vulgaris* spermiogenesis during the crucial stage of somatic to generative nucleus rearrangement. The accumulation of CRT, particularly at nuclear peripheries containing condensed chromatin, indicates its involvement in the translocation of newly synthesized protamines (Popłońska 2013). Recent studies by Liu et al. (2022) provide compelling evidence for the translocation of CRT into the cell nucleus, linking this process with apoptosis. Histone deacetylase inhibitors (HDACis) induced both CRT translocation and Ca^2+^ accumulation in hepatocarcinoma cell nuclei, leading to the attenuation of the CaM/CaMKII/CREB signaling pathway and inducing apoptotic events. These observations confirm that disruptions in Ca^2+^ signaling mechanisms may lead to the destabilization of cellular homeostasis and ultimately to the cell death (Liu et al. 2022). In the context of our results, linking CRT translocation to the nucleus with progressive apoptotic processes within the transmitting tissue following fertilization is a tempting proposition. Furthermore, some reports support the impact of CRT, localized in the nuclear envelope, on the structure of the nuclear pore complex. In vitro studies using CRT knockout cardiomyocytes confirm a disturbed structure and function of nuclear pores in Ca^2+^-dependent manner (Faustino et al. 2016). This possibility is linked with Ca^2+^-dependent signaling within the nucleus, as well as nucleocytoplasmic transport. Gomes et al. (2006) described connections of CRT with reticular structures as an extension of the ER and located in sub-nuclear regions. According to this thesis, the structures, called nuclear reticulum, play a role as Ca^2+^ reservoirs necessary for signaling pathways and transcription process (Gomes et al. 2006; Mazars et al. 2011). Additionally, other data show that the import of the MEF2C transcription factor is disrupted in mouse embryonic stem cells with CRT’s deficit (Faustino et al. 2016). Also, CRT was noted to participate in the export of nuclear hormone receptors, especially steroid, non-steroid, and orphan receptors (Roderick et al. 1997; Holaska et al. 2001; Brünagel et al. 2003).

In conclusion, our study contributes additional evidence supporting the presence of CRT in the cell nucleus, affirming observations previously proposed by various research teams. This research demonstrates the consistent presence of CRT, along with exchangeable Ca^2+^, within defined domains of the nuclei of highly specialized plant cells, specifically the cells of the pistil transmitting tract. Notably, this observation holds true irrespective of the anatomical structure and the evolutionary distance of different plant species.

## Supplementary Information

Below is the link to the electronic supplementary material.

Supplementary file1 **Negative control (a, b) and specificity control of a maize CRT PAb (c).** of CRT in pSty of *Petunia* (a) and *Haemanthus* (b). *No nucleolus*, *Nu nucleus*, *Ttc transmitting tissue cells*, *Tte transmitting tissue epidermis. Bars* 200 nm. (c) Western blotting of the total protein extracts from *Z.mays* (lane 1), *P.hybrida* (lane 2) and *H.albiflos* (lane 3). The protein marker (Protein Marker VI, 10 – 245, prestained, AppliChem) is indicated on the left. (PNG 381 KB)

Supplementary file2 **Immunogold distribution of CRT in *Petunia* (a-c) and *Haemanthus* (d-f) styles before and after pollination using commercial CRT PAb.** Localization of CRT in *Petunia* styles before (a) and after pollination (b-c). Distribution of CRT in *Haemanthus* uSty and pSty (d and e-f, respectively). *No nucleolus*, *Nu nucleus*, *Ttc transmitting tissue cells*, *Tte* transmitting tissue epidermis. *Bars* 500 nm. (g) Immunoblot of soluble protein fractions from *M.musculus* (lane 1), *P.hybrida* (lane 2) and *H.albiflos* (lane 3). The protein marker (Protein Marker VI, 10 – 245, prestained, AppliChem) is indicated on the left. (PNG 1257 KB)

Supplementary file3 **Immunogold localization of CNX using commercial CNX1/2 PAb (a-c) in *Petunia* styles before and after pollination.** Distribution of CNX in uSty (a) and pSty (b) of *Petunia*. Negative control reaction for pSty (c). *Cy* cytosol, *Er* endoplasmic reticulum, *No nucleolus*, *Nu* nucleus. *Bars* 200 nm. (d) Immunoblotting of the total protein extracts from *A.thaliana* (lane 1), *P.hybrida* (lane 2) and *H.albiflos* (lane 3). The protein marker (PageRuler™ Prestained Protein Ladder, 10-180, Thermo Scientific) is indicated on the left. (PNG 896 KB)

## Abbreviations

Ca^2+^: Calcium/calcium ions
Ca^2+^ ppts: Ca^2+^-antimonate precipitates
*CRT*: Calreticulin gene
CRT: Calreticulin protein
CRT PAb: Polyclonal antibody against CRT
ER: Endoplasmic reticulum
HDEL: ER-retention sequence of plant CRT
NLS: Nuclear localization signal
pSti: Pollinated stigma
pSty: Pollinated style
uSti: Unpollinated stigma
uSty: Unpollinated style
