# Supplementary figures and images for: Plant-specific calreticulin is localized in the nuclei of highly specialized cells in the pistil—new observations for an old hypothesis
Source: Protoplasma. 2024 Jun 7;261(6):1171–84. doi: 10.1007/s00709-024-01961-y (PMC11511736; doi:10.1007/s00709-024-01961-y)

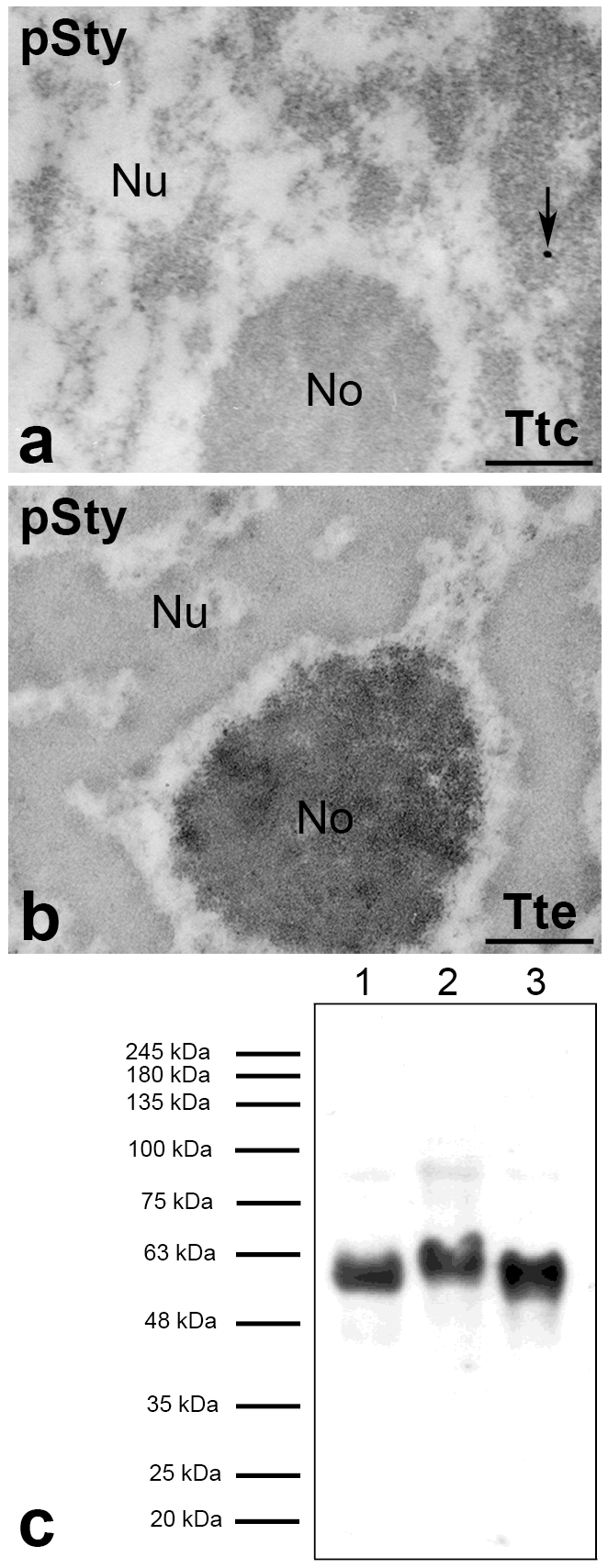

Supplement: Supplementary file 1 — Supplementary file1 Negative control (a, b) and specificity control of a maize CRT PAb (c). of CRT in pSty of Petunia (a) and Haemanthus (b). No nucleolus, Nu nucleus, Ttc transmitting tissue cells, Tte transmitting tissue epidermis. Bars 200 nm. (c) Western blotting of the total protein extracts from Z.mays (lane 1), P.hybrida (lane 2) and H.albiflos (lane 3). The protein marker (Protein Marker VI, 10 – 245, prestained, AppliChem) is indicated on the left. (PNG 381 KB) [file 709_2024_1961_MOESM1_ESM.png]

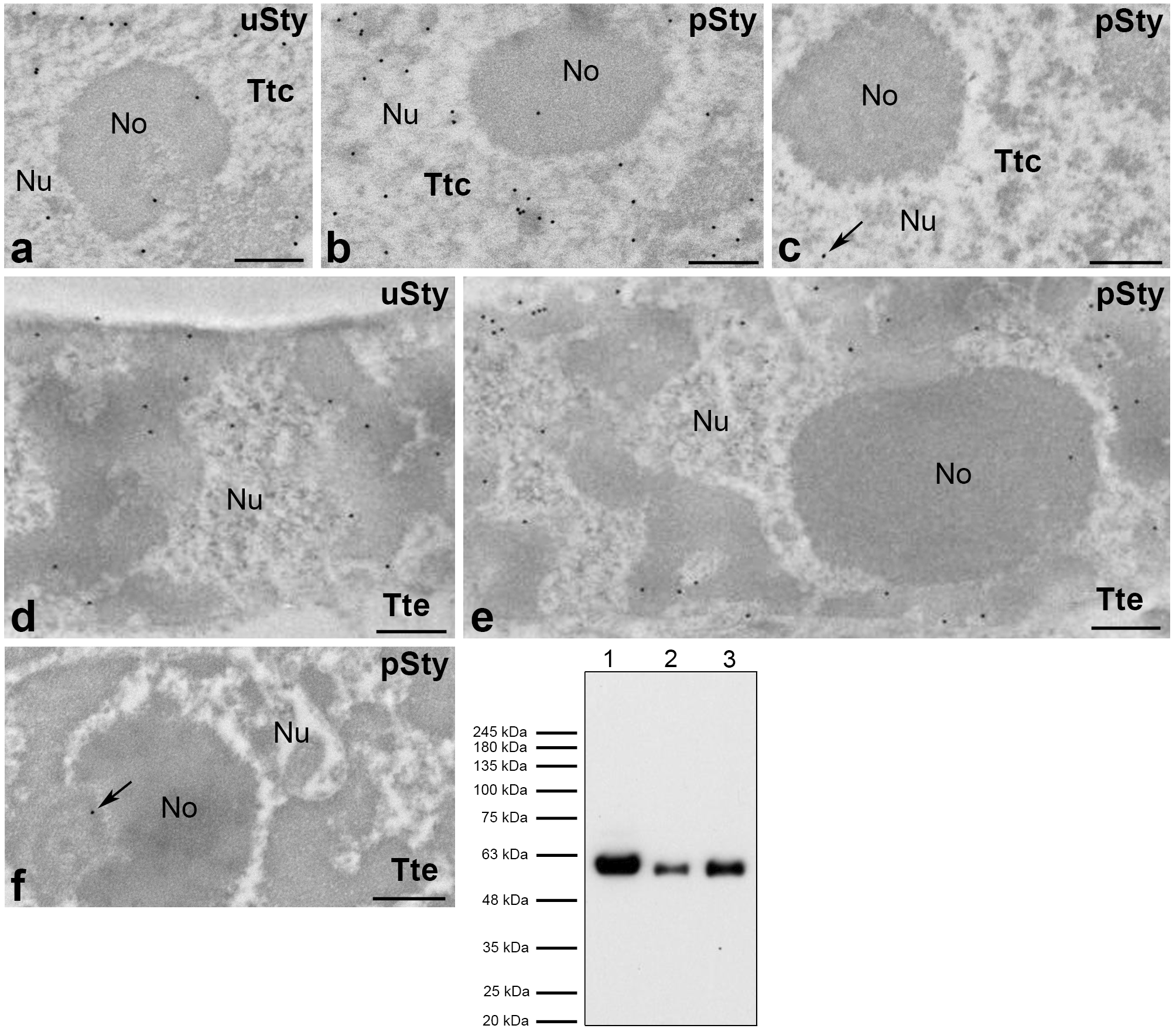

Supplement: Supplementary file 2 — Supplementary file2 Immunogold distribution of CRT in Petunia (a-c) and Haemanthus (d-f) styles before and after pollination using commercial CRT PAb. Localization of CRT in Petunia styles before (a) and after pollination (b-c). Distribution of CRT in Haemanthus uSty and pSty (d and e-f, respectively). No nucleolus, Nu nucleus, Ttc transmitting tissue cells, Tte transmitting tissue epidermis. Bars 500 nm. (g) Immunoblot of soluble protein fractions from M.musculus (lane 1), P.hybrida (lane 2) and H.albiflos (lane 3). The protein marker (Protein Marker VI, 10 – 245, prestained, AppliChem) is indicated on the left. (PNG 1257 KB) [file 709_2024_1961_MOESM2_ESM.png]

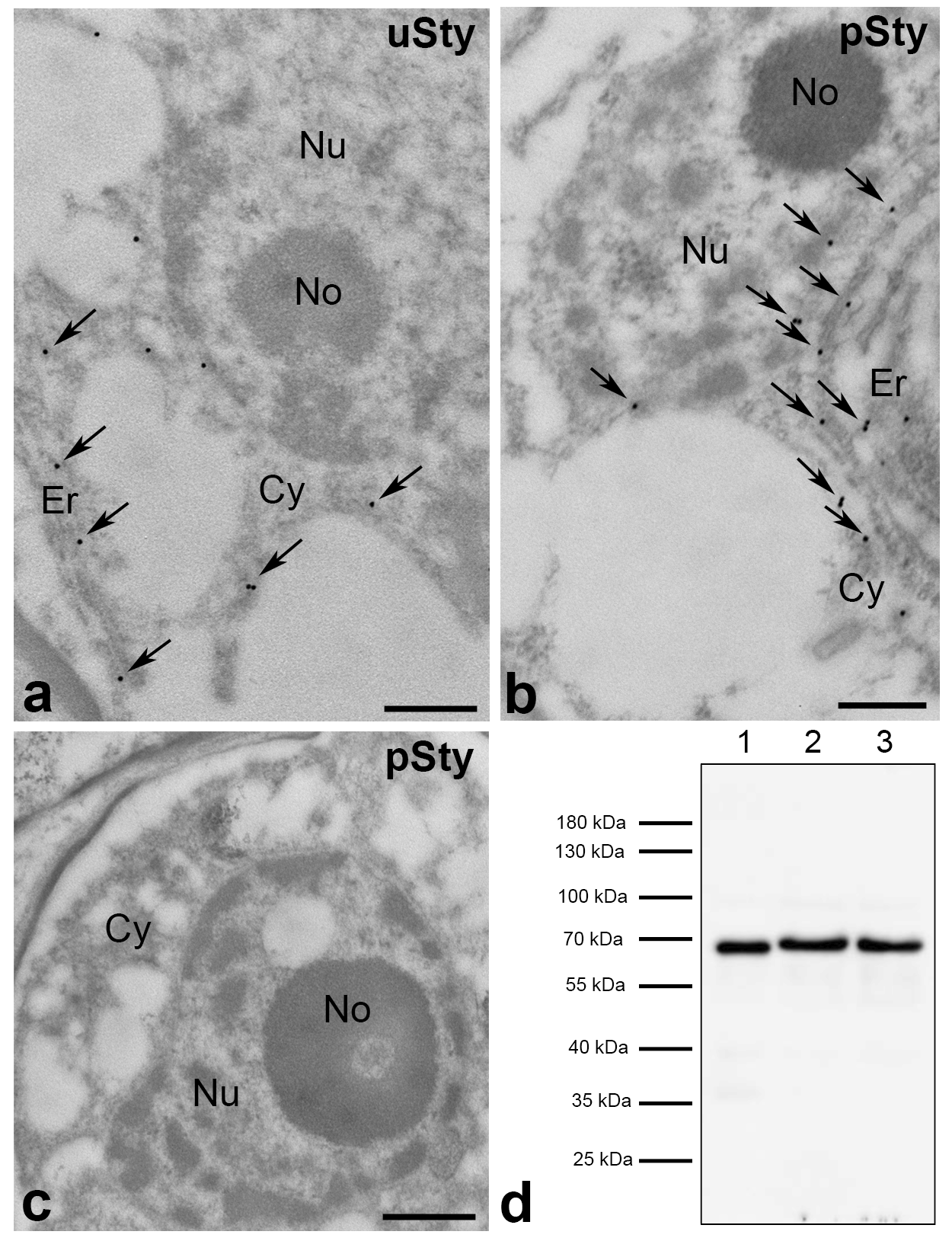

Supplement: Supplementary file 3 — Supplementary file3 Immunogold localization of CNX using commercial CNX1/2 PAb (a-c) in Petunia styles before and after pollination. Distribution of CNX in uSty (a) and pSty (b) of Petunia. Negative control reaction for pSty (c). Cy cytosol, Er endoplasmic reticulum, No nucleolus, Nu nucleus. Bars 200 nm. (d) Immunoblotting of the total protein extracts from A.thaliana (lane 1), P.hybrida (lane 2) and H.albiflos (lane 3). The protein marker (PageRuler™ Prestained Protein Ladder, 10-180, Thermo Scientific) is indicated on the left. (PNG 896 KB) [file 709_2024_1961_MOESM3_ESM.png]
